# A 9 year retrospective review of motorcycle accidents at a level 1 trauma center in Riyadh

**DOI:** 10.1016/j.sopen.2025.09.006

**Published:** 2025-09-09

**Authors:** Hussam Alhathlol, Khalid Alsikhan, Turki Alharbi, Ibrahim Alsamaani, Ali Alhathloul, Rifan Alyami

**Affiliations:** a College of Medicine, King Saud bin Abdulaziz University for Health Sciences, Riyadh, Saudi Arabia; b Department of Surgery, King Abdulaziz Medical City, Saudi Arabia

**Keywords:** Motorcycle accidents, Injury patterns, Fractures, Brain injuries, Surgical intervention

## Abstract

**Introduction:**

Motorcycle accidents are a significant cause of morbidity and mortality worldwide, with an increasing trend observed in Saudi Arabia. These accidents often result in severe injuries, leading to long-term disability or death, highlighting the need for better understanding and management in trauma centers. These findings highlight the vulnerability of motorcyclists on the roads and underscore the critical need to address motorcycle safety in order to reduce the burden of road traffic accidents.

**Methods:**

This retrospective study assessed the rate, injury patterns, and outcomes of motorcycle accidents at King Abdulaziz Medical City (KAMC) in Riyadh, a level 1 trauma center, from January 2016 to December 2024. A total of 415 adult patients were included, with data on demographics, injury types, and treatment outcomes collected from hospital records. Multivariable logistic regression was performed to identify predictors of intensive care unit (ICU) admission, intubation, and surgical intervention.

**Results:**

The cohort consisted predominantly of young males (84.1 %), with the highest incidence observed in the 18–35 age group. Common injuries included fractures (86.7 %), brain injuries/bleeding (38.1 %), and cut/open wounds (27.5 %). Although the incidence peaked in 2023, no statistically significant trend was observed over the study period. Moreover, a surgical intervention was required in 69.9 % of the cases, with 28.2 % experiencing long-term disability, which was defined based on discharge disposition and documented rehabilitation needs Factors significantly associated with ICU admission and intubation included head injuries, chest injuries, and facial trauma.

**Conclusion:**

Motorcycle accidents continue to pose a significant public health challenge in Saudi Arabia, with young male motorcyclists being the most vulnerable group. The high incidence of fractures and brain injuries emphasizes the importance of improving safety measures to reduce the severity of injuries.

## Introduction

Road traffic injuries are a very common and critical cause of morbidity and mortality around the world [1]. Globally, road traffic crashes cause approximately 1.19 million deaths annually according to the World Health Organization [2]. Moreover, road traffic crashes cost most countries 3 % of their gross domestic product causing significant economic burden [2,3]. In Saudi Arabia, road injuries are ranked the third leading cause of death causing 36 deaths per 100,000 population [4]. More recent statistics from the Saudi Ministry of Health (MOH) report a rising number of road traffic injuries, with 76 injuries per 100,000 population [5].

While there have been numerous studies assessing the broader spectrum of motor vehicle accidents in Saudi Arabia, motorcycle accidents have received relatively less attention in comparison [[6], [7], [8], [9], [10]]. Motorcyclists face a significantly higher risk of fatality and injury compared to occupants of passenger cars in the event of a crash. Previous studies have found that motorcyclists are around 8 times more likely to sustain injuries and 35 times more likely to cause death when involved in a collision [11]. This focus is critical because motorcyclists face a disproportionate risk of harm. These injuries are also associated with a high economic burden due to the need for surgical care, intensive care, and long-term rehabilitation [3]. Regarding fatalities of motorcycle accidents, there has been a 2 % increase in 2023 compared to 2022 in the US [12]. Locally there are no statistics that describe motorcycle accident trends in recent years.

Motorcycles have become an increasingly popular mode of transportation in Saudi Arabia because they offer a convenient and maneuverable option for navigating congested roads. Particularly, online food ordering applications have increased in popularity in the post-COVID period, and these applications employ motorcycle riders to deliver. However, this growth in motorcycle usage has also been accompanied by a concerning rise in the number of motorcycle-related accidents and fatalities. Therefore, we aim to assess the rate, injury pattern, and outcomes of motorcycle accidents to provide insights that can inform policies and interventions aimed at reducing the burden of motorcycle-related accidents.

## Methods

The study was conducted at King Abdulaziz Medical City, Riyadh. KAMC is a level 1 trauma center with 24/7 availability of specialized care. The center is equipped to handle all types of traumatic injuries, including complex cases such as multiple traumas, severe head injuries, and spinal cord injuries. The center is also home to a trauma registry, which is a comprehensive database capturing all patients admitted following traumatic events, including motorcycle accidents. The study was conducted at a single trauma center, which may limit generalizability to other regions.

The study included all adult patients admitted following a motorcycle accident from January 2016 to December 2024. On the other hand, patients with minor injuries not necessitating hospital admission were excluded. Additionally, patients admitted for non-trauma related reasons, out of hospital deaths or transferred from other hospitals were also excluded.

The study was approved by the institutional review board (IRB) at KAIMRC protocol no. NRR24/080/7 as a retrospective review, informed consent was not required. All patient data were anonymized to maintain confidentiality and privacy in accordance with ethical standards.

Demographic data, including age, sex, nationality, and body mass index (BMI), were collected for each patient. The primary measures of interest included injury type and severity, need for surgical intervention, intensive care unit (ICU) admission, intubation, or prolonged hospital stay, mortality rate, long-term disability and rehabilitation needs, the use of safety measures (e.g., helmets, protective gear), and injury locations and specific body areas affected (head, limbs, chest, abdomen, etc.).

Injury types were categorized based on documentation in the trauma registry, which is derived from clinical notes and documented by trained registry staff. Data on helmet use were also extracted from trauma registry entries, which rely on documentation by emergency department physicians.

All statistical analyses were performed using RStudio (version 2024.9.1.394, Boston, MA, USA, using R version 4.4.2). Categorical variables were analyzed using Fisher's exact test or Pearson's chi-squared test as appropriate. Binary logistic regression models were constructed to examine factors associated with specific outcomes related to motorcycle accidents. Variables with a *p*-value of less than 0.05 in the univariate analyses were included in the multivariable logistic regression models to adjust for potential confounders. A *p*-value of <0.05 was considered statistically significant.

Missing data for BMI (1 %, 4 records) were excluded from subgroup analyses. For safety measures, where 91.1 % of cases were undocumented (Unknown), these cases were reported descriptively as a separate category, but excluded from multivariable regression.

## Results

### Demographic characteristics and incidence over time

The current study included data of 415 patients. The majority of patients were male (84.1 %), and the most common age groups were 25 to 35 years (38.6 %) and 18 to 25 years (36.6 %). Regarding BMI, 37.5 % had a healthy BMI, followed by 29.9 % who were overweight and 25.5 % who were obese. Saudi nationals accounted for 68.9 % of the cases, while non-Saudis comprised 31.1 % (Table 1).

**Table 1:** Demographic characteristics and the statistical differences in the incidence of motorcycle accidents over time.

Characteristic	Overall N = 415	2016 N = 41	2017 N = 41	2018 N = 55	2019 N = 54	2020 N = 36	2021 N = 38	2022 N = 48	2023 N = 62	2024 N = 40	p-Value
Age											0.247
18 to <25	152 (36.6 %)	20 (48.8 %)	18 (43.9 %)	25 (45.5 %)	23 (42.6 %)	14 (38.9 %)	8 (21.1 %)	14 (29.2 %)	18 (29.0 %)	12 (30.0 %)	
25 to <35	160 (38.6 %)	15 (36.6 %)	12 (29.3 %)	18 (32.7 %)	19 (35.2 %)	15 (41.7 %)	18 (47.4 %)	19 (39.6 %)	26 (41.9 %)	18 (45.0 %)	
35 to <45	69 (16.6 %)	4 (9.8 %)	6 (14.6 %)	6 (10.9 %)	10 (18.5 %)	5 (13.9 %)	4 (10.5 %)	12 (25.0 %)	13 (21.0 %)	9 (22.5 %)	
45 to <55	26 (6.3 %)	1 (2.4 %)	5 (12.2 %)	5 (9.1 %)	1 (1.9 %)	1 (2.8 %)	5 (13.2 %)	3 (6.3 %)	4 (6.5 %)	1 (2.5 %)	
55 to <65	8 (1.9 %)	1 (2.4 %)	0 (0.0 %)	1 (1.8 %)	1 (1.9 %)	1 (2.8 %)	3 (7.9 %)	0 (0.0 %)	1 (1.6 %)	0 (0.0 %)	
Gender											0.038
Male	349 (84.1 %)	32 (78.0 %)	38 (92.7 %)	51 (92.7 %)	39 (72.2 %)	27 (75.0 %)	33 (86.8 %)	39 (81.3 %)	54 (87.1 %)	36 (90.0 %)	
Female	66 (15.9 %)	9 (22.0 %)	3 (7.3 %)	4 (7.3 %)	15 (27.8 %)	9 (25.0 %)	5 (13.2 %)	9 (18.8 %)	8 (12.9 %)	4 (10.0 %)	
BMI^a^											0.748
Underweight	29 (7.1 %)	2 (5.1 %)	3 (7.3 %)	1 (1.9 %)	3 (5.6 %)	2 (5.6 %)	3 (7.9 %)	6 (12.5 %)	6 (9.7 %)	3 (7.5 %)	
Healthy	154 (37.5 %)	12 (30.8 %)	15 (36.6 %)	23 (43.4 %)	26 (48.1 %)	14 (38.9 %)	9 (23.7 %)	18 (37.5 %)	20 (32.3 %)	17 (42.5 %)	
Overweight	123 (29.9 %)	12 (30.8 %)	14 (34.1 %)	19 (35.8 %)	11 (20.4 %)	11 (30.6 %)	11 (28.9 %)	13 (27.1 %)	22 (35.5 %)	10 (25.0 %)	
Obese	105 (25.5 %)	13 (33.3 %)	9 (22.0 %)	10 (18.9 %)	14 (25.9 %)	9 (25.0 %)	15 (39.5 %)	11 (22.9 %)	14 (22.6 %)	10 (25.0 %)	
Nationality											0.239
Saudi	286 (68.9 %)	28 (68.3 %)	32 (78.0 %)	41 (74.5 %)	42 (77.8 %)	26 (72.2 %)	26 (68.4 %)	29 (60.4 %)	40 (64.5 %)	22 (55.0 %)	
Non-Saudi	129 (31.1 %)	13 (31.7 %)	9 (22.0 %)	14 (25.5 %)	12 (22.2 %)	10 (27.8 %)	12 (31.6 %)	19 (39.6 %)	22 (35.5 %)	18 (45.0 %)	

n (%).

Fisher's exact test; Pearson's Chi-squared test.

a BMI values had 4 missing records.

**Fig. 1:**
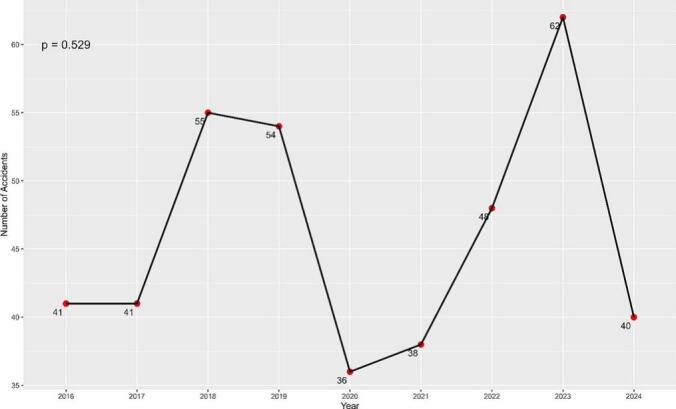
The frequencies of motorcycle accidents over time.

The number of motorcycle accidents remained stable in 2016 and 2017 (*n* = 41 each year), followed by a slight increase in 2018 and 2019 (*n* = 55 and 54, respectively). A noticeable decline occurred in 2020, with the lowest number of accidents reported during the study period (*n* = 36). Subsequently, the frequency increased gradually, reaching 48 in 2022 and peaking at 62 in 2023, before decreasing again to 40 in 2024. Despite these fluctuations, the Mann-Kendall test indicated no statistically significant trend in accident incidence over the study period (*p* = 0.529, Fig. 1). The peak in 2023 may reflect contextual factors such as the expansion of motorcycle-based delivery services in Riyadh. Though specific data to confirm this was unavailable.

Regarding the statistical differences, only gender showed a statistically significant difference in the incidence of motorcycle accidents over time (*p* = 0.038). Males consistently accounted for the majority of cases each year, ranging from 72.2 % in 2019 to 92.7 % in 2017 and 2018 (Table 1).

### Characteristics of injuries and outcomes

Most accidents occurred on streets or roads (90.8 %), with unknown activities reported in 93.7 % of cases. The use of safety measures was reported in only 2.7 % of cases, while 6.3 % had no safety measures. The most common injury types were fractures (86.7 %), brain injuries/bleeding (37.6 %), and cut/open wounds (27.5 %). Regarding outcomes, 64.8 % of patients did not require ICU admission, and 70.8 % did not undergo intubation. However, 69.9 % required surgical intervention, and hospital stays were typically less than one month (88.0 %). Long-term disability, which was defined according to discharge disposition and recorded rehabilitation requirements, was reported in 28.2 % of cases, and mortality occurred in 4.4 % (Table 2).

**Table 2:** Characteristics of injuries and outcomes of patients.

Characteristic	Description
Activity at time of injury	
Leisure	22 (5.3 %)
Work	2 (0.5 %)
Unknown	389 (93.7 %)
Others	2 (0.5 %)
Where did the injury occur	
Street/road	377 (90.8 %)
Chalet/resort/camping	35 (8.4 %)
Others	3 (0.7 %)
Presence of safety measures	
No	26 (6.3 %)
Yes	11 (2.7 %)
Unknown	378 (91.1 %)
Multiple injuries	182 (43.9 %)
GCS	
0 to 3	33 (8.0 %)
4 to 7	15 (3.6 %)
8 to 9	41 (9.9 %)
10 to 12	24 (5.8 %)
12 to 15	302 (72.8 %)
ICU length of stay (weeks)	
None	269 (64.8 %)
<2	94 (22.7 %)
2 to 4	38 (9.2 %)
4 to 6	11 (2.7 %)
6 to 8	3 (0.7 %)
Intubation period (weeks)	
None	294 (70.8 %)
<2	99 (23.9 %)
2 to 4	22 (5.3 %)
Need for surgical intervention	
No	124 (29.9 %)
Yes	290 (69.9 %)
Unknown	1 (0.2 %)
Hospital length of stay (months)	
<1	365 (88.0 %)
1 to 3	32 (7.7 %)
3 to 6	10 (2.4 %)
6 to 9	3 (0.7 %)
9 to 12	3 (0.7 %)
>12	2 (0.5 %)
Hospital disposition and projected effect of injury^a^	
No significant disability	139 (33.8 %)
Short-term disability	113 (27.5 %)
Long-term disability	116 (28.2 %)
Permanent disability	25 (6.1 %)
Death	18 (4.4 %)

n (%).

GCS = Glascow Coma Scale.

ICU = intensive care unit.

a The variable had 4 missing records.

**Fig. 2:**
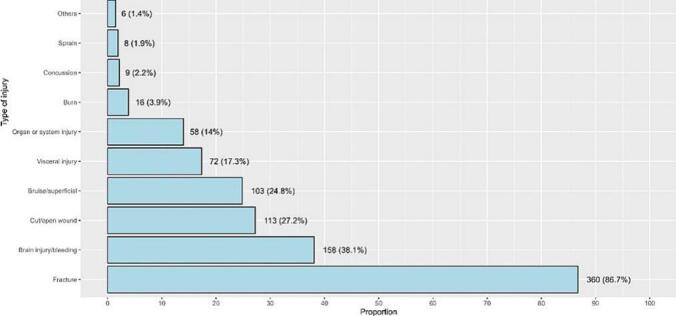
Types of injuries after motorcycle accidents.

**Fig. 3:**
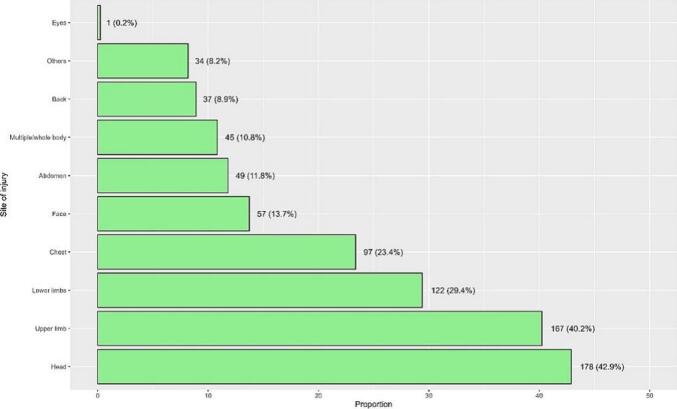
Sites of injuries after motorcycle accidents.

Fig. 2 illustrates the distribution of injury types among patients involved in motorcycle accidents. Fractures were the most common injury, affecting 86.7 % of patients, followed by brain injuries or bleeding (38.1 %) and cut/open wounds (27.2 %). Regarding injury sites, injuries to the head and upper limbs were the most prevalent, affecting 42.9 % and 40.2 % of patients, respectively, followed by lower limb injuries (29.4 %) and chest injuries (23.4 %, Fig. 3).

### Factors associated with surgical intervention

The likelihood of undergoing a surgical intervention was significantly increased with specific injury types, including cut/open wounds (*p* = 0.005), lower limb injuries (*p* < 0.001), and upper limb injuries (p < 0.001) (Table 3).

**Table 3:** Factors associated with the need for surgical interventions.

Characteristic	Inferential analysis			Multivariable regression		
	No *N* = 125	Yes *N* = 290	p-Value	OR	95 % CI	p-Value
Age			0.756			
18 to <25	44 (28.9 %)	108 (71.1 %)				
25 to <35	50 (31.3 %)	110 (68.8 %)				
35 to <45	20 (29.0 %)	49 (71.0 %)				
45 to <55	7 (26.9 %)	19 (73.1 %)				
55 to <65	4 (50.0 %)	4 (50.0 %)				
Sex			0.797			
Male	106 (30.4 %)	243 (69.6 %)				
Female	19 (28.8 %)	47 (71.2 %)				
Presence of safety measures			0.177			
No	11 (42.3 %)	15 (57.7 %)				
Yes	5 (45.5 %)	6 (54.5 %)				
Unknown	109 (28.8 %)	269 (71.2 %)				
Type of injury						
Brain injury bleeding	68 (43.6 %)	88 (56.4 %)	<0.001	2.09	0.80, 5.76	0.140
Bruise superficial	31 (30.1 %)	72 (69.9 %)	0.995			
Fracture	92 (25.6 %)	268 (74.4 %)	<0.001	1.59	0.78, 3.26	0.200
Visceral injury	30 (40.0 %)	45 (60.0 %)	0.039	0.97	0.48, 1.97	0.924
Organ or system injury	28 (47.5 %)	31 (52.5 %)	0.002	0.76	0.35, 1.61	0.466
Open wound	25 (21.9 %)	89 (78.1 %)	0.025	2.51	1.34, 4.89	0.005
Concussion	5 (55.6 %)	4 (44.4 %)	0.136			
Burn	2 (12.5 %)	14 (87.5 %)	0.165			
Sprain	1 (12.5 %)	7 (87.5 %)	0.445			
Others	3 (50.0 %)	3 (50.0 %)	0.371			

n (%).

Fisher's exact test; Pearson's Chi-squared test.

OR = odds ratio, CI = confidence interval.

The multivariable regression model was based on binomial logistic regression analysis, which included the significantly associated variables from the inferential analysis as independent variables.

### Factors associated with ICU admission

ICU admission was associated with sex, with females less likely to require ICU care than males (*p* = 0.048). Brain injuries/bleeding were significant risk factors for ICU admission (*p* = 0.007). Having injuries in specific sites was significantly associated with an increased odds of ICU admission, including head injuries (*p* < 0.001), chest injuries (*p* = 0.012) and whole-body injuries (p < 0.001, Table 4).

**Table 4:** Factors associated with ICU admission.

Characteristic	Inferential analysis			Multivariable regression		
	No *N* = 269	Yes *N* = 146	p-Value	OR	95 % CI	p-Value
Age			0.791			
18 to <25	94 (61.8 %)	58 (38.2 %)				
25 to <35	106 (66.3 %)	54 (33.8 %)				
35 to <45	44 (63.8 %)	25 (36.2 %)				
45 to <55	19 (73.1 %)	7 (26.9 %)				
55 to <65	6 (75.0 %)	2 (25.0 %)				
Sex			<0.001			
Male	211 (60.5 %)	138 (39.5 %)		Reference	Reference	
Female	58 (87.9 %)	8 (12.1 %)		0.36	0.12, 0.97	0.048
Type of injury						
Brain injury bleeding	40 (25.6 %)	116 (74.4 %)	<0.001	3.74	1.45, 10.0	0.007
Bruise superficial	68 (66.0 %)	35 (34.0 %)	0.769			
Fracture	237 (65.8 %)	123 (34.2 %)	0.268			
Visceral injury	23 (30.7 %)	52 (69.3 %)	<0.001	2.46	0.99, 6.24	0.054
Organ or system injury	18 (30.5 %)	41 (69.5 %)	<0.001	2.15	0.80, 6.05	0.138
Cut open wound	55 (48.2 %)	59 (51.8 %)	<0.001	1.64	0.76, 3.56	0.207
Concussion	3 (33.3 %)	6 (66.7 %)	0.072			
Burn	9 (56.3 %)	7 (43.8 %)	0.464			
Others	6 (100.0 %)	0 (0.0 %)	0.095			
Sprain	7 (87.5 %)	1 (12.5 %)	0.270			
Site of injury						
Abdomen	14 (28.6 %)	35 (71.4 %)	<0.001	1.57	0.46, 5.50	0.473
Back	14 (38.9 %)	22 (61.1 %)	<0.001	1.14	0.33, 4.23	0.835
Head	47 (28.5 %)	118 (71.5 %)	<0.001	9.84	3.34, 29.9	<0.001
Chest	28 (30.4 %)	64 (69.6 %)	<0.001	3.08	1.30, 7.59	0.012
Lower limbs	76 (63.9 %)	43 (36.1 %)	0.796			
Face	12 (21.1 %)	45 (78.9 %)	<0.001	2.85	1.17, 7.33	0.024
Others	14 (42.4 %)	19 (57.6 %)	0.005	1.81	0.61, 5.51	0.289
Upper limb	131 (79.4 %)	34 (20.6 %)	<0.001	0.55	0.24, 1.22	0.144
Multiple whole body	23 (51.1 %)	22 (48.9 %)	0.041	6.52	2.18, 20.5	<0.001
Eyes	0 (0.0 %)	1 (100.0 %)	0.352			

n (%).

Fisher's exact test; Pearson's Chi-squared test.

OR = odds ratio, CI = confidence interval.

The multivariable regression model was based on binomial logistic regression analysis, which included the significantly associated variables from the inferential analysis as independent variables.

### Factors associated with intubation

Table 5 presents factors associated with intubation among patients involved in motorcycle accidents. Brain injuries/bleeding (*p* = 0.020) and head injuries (p < 0.001) were significantly associated with higher odds of intubation. Additionally, injuries to the face (*p* = 0.003) and other sites (p = 0.020) were also significantly linked to intubation.

**Table 5:** Factors associated with intubation.

Characteristic	Inferential analysis			Multivariable regression		
	No *N* = 294	Yes *N* = 121	p-Value	OR	95 % CI^3^	p-Value
Age			0.950			
18 to <25	109 (71.7 %)	43 (28.3 %)				
25 to <35	114 (71.3 %)	46 (28.8 %)				
35 to <45	46 (66.7 %)	23 (33.3 %)				
45 to <55	19 (73.1 %)	7 (26.9 %)				
55 to <65	6 (75.0 %)	2 (25.0 %)				
Gender			<0.001			
Male	232 (66.5 %)	117 (33.5 %)		Reference	Reference	
Female	62 (93.9 %)	4 (6.1 %)		0.17	0.04, 0.53	0.004
Type of injury						
Brain injury bleeding	56 (35.9 %)	100 (64.1 %)	<0.001	3.01	1.18, 7.73	0.020
Bruise superficial	69 (67.0 %)	34 (33.0 %)	0.321			
Fracture	257 (71.4 %)	103 (28.6 %)	0.532			
Visceral injury	33 (44.0 %)	42 (56.0 %)	<0.001	1.53	0.63, 3.71	0.347
Organ or system injury	26 (44.1 %)	33 (55.9 %)	<0.001	1.24	0.52, 3.04	0.628
Cut open wound	64 (56.1 %)	50 (43.9 %)	<0.001	1.87	0.89, 3.94	0.097
Concussion	4 (44.4 %)	5 (55.6 %)	0.130			
Burn	13 (81.3 %)	3 (18.8 %)	0.416			
Others	5 (83.3 %)	1 (16.7 %)	0.676			
Site of injury						
Abdomen	19 (38.8 %)	30 (61.2 %)	<0.001	2.19	0.73, 6.91	0.168
Back	19 (52.8 %)	17 (47.2 %)	0.013	0.55	0.18, 1.67	0.292
Head	62 (37.6 %)	103 (62.4 %)	<0.001	7.15	2.66, 19.8	<0.001
Chest	40 (43.5 %)	52 (56.5 %)	<0.001	1.71	0.75, 3.93	0.203
Lower limbs	83 (69.7 %)	36 (30.3 %)	0.756			
Face	16 (28.1 %)	41 (71.9 %)	<0.001	3.59	1.58, 8.53	0.003
Others	14 (42.4 %)	19 (57.6 %)	<0.001	3.65	1.24, 11.1	0.020
Upper limb	138 (83.6 %)	27 (16.4 %)	<0.001	0.52	0.23, 1.12	0.099
Multiple whole body	29 (64.4 %)	16 (35.6 %)	0.317			
Eyes	0 (0.0 %)	1 (100.0 %)	0.292			

n (%).

Fisher's exact test; Pearson's Chi-squared test.

OR = odds ratio, CI = confidence interval.

The multivariable regression model was based on binomial logistic regression analysis, which included the significantly associated variables from the inferential analysis as independent variables.

## Discussion

This nine-year retrospective review demonstrates the significant burden of motorcycle-related injuries in Saudi Arabia, with severe consequences including long-term disability and death. As our study showed fractures were the most prevalent injury affecting 86.7 % of patients, reflecting the high-impact nature of motorcycle crashes and the absence of protective barriers compared to enclosed vehicles. Brain injuries or bleeding were the second most common injuries, affecting 38.1 % of patients, which is concerning given the high mortality and long-term disability associated with such injuries. Cut and open wounds were seen in 27.2 % of patients, reflecting the severity of road crash and external injuries are common in motorcycle accidents. The head and upper limbs were the most commonly injured sites, with significant associations between these injuries and the need for ICU care or surgery. Head injuries were particularly associated with severe outcomes, including ICU admission, intubation, and surgical intervention.

The majority of the patients were male (84.1 %), with the highest incidence observed in the age groups 18–25 and 25–35 years, likely due to the increased use of motorcycles in these age groups. The study also notes an increase in motorcycle accidents, peaking in 2023, although no statistically significant trend over time was found. The significant decrease in accidents in 2020 (*n* = 36) can likely be attributed to the direct effects of the COVID-19 pandemic, which resulted in lockdowns, curfews, and restrictions on travel, leading to fewer motorcycles on the road.

Our study's main findings on the impact of motorcycle accidents are consistent with many other publications. Various studies [11,13,14] reported similar age and gender proportions, with the average age ranging from 21 to 32 and a constant predominant male proportion of 85 % to 86 % across these studies. In our trauma center, Suliman Alghnam et al [11] reported similar demographic results with male predominance of 85 % with the great majority of patients being younger than 40 years old and clustered around the 20s range. It is very important to note that these were pre-COVID-19 findings, pointing out that the pandemic did not influence the main demographic trends seen across studies.

Our data also revealed that more than half of riders were overweight or obese. While current evidence suggests BMI is not directly associated with mortality or hospital length of stay [15], biomechanics may differ, greater body mass increases kinetic energy transfer during crashes, potentially predisposing to specific fracture patterns or higher injury severity. Future studies could explore how body composition influences injury mechanisms in motorcycle accidents.

The presence of safety measures was only reported in 9 % of the patients in our study, which means that documentation is still an issue being faced in our center, as also reported by Suliman Alghnam et al [11] in his study in 2019. This poor documentation likely stems from factors such as inconsistent reporting by emergency department physicians. Furthermore, the absence of mandatory fields for safety measures in the KAMC trauma registry likely exacerbates this issue. Wearing a helmet and following safety protocols while riding a motorcycle are mandatory traffic regulations in Saudi Arabia. Therefore, we would expect a high level of compliance with these regulations among most patients, which may contribute to a predominance of mild to moderate injuries, as observed across various variables in Table 3. This is consistent with findings from other studies that have linked the severity of injuries to the use of safety measures [19].

Extremity injuries (69.6 %) were the most common site of injury in our study, followed by head (42.2 %) and chest injuries (23.4 %), which is a trend observed and expected with some variation in almost all motorcycle accidents studies [11,[13], [14], [15], [16], [17]]. Surgical interventions mostly were needed for orthopedic injuries which may include open fractures and open wound injuries. Fletcher et al published a study that focused on predictors of hospitalization and surgical intervention, and most patients that required either or both were orthopedic patients [18]. ICU admission and intubation, on the other hand, was observed more in the head injury spectrum compared to fractures or extremity injuries, which, for the latter, is not usually severe to require ICU admission. This pattern was also noticed in other studies that assessed the severity of head injuries in motorcycle accidents [20].

Given the high incidence of fractures and brain injuries observed in this study, enhanced protective measures such as stricter helmet enforcement and safety training programs could significantly reduce morbidity and mortality. Studies have shown that helmet use can reduce the risk of severe head injuries by 69 % and decrease the likelihood of fatal outcomes by 42 % [21].

Moreover, the substantial proportion of patients requiring surgical intervention (69.9 %) increases the burden on healthcare resources, emphasizing the necessity of optimizing trauma care infrastructure, including orthopedic and neurosurgical services. A study in the United States found that motorcycle-related injuries accounted for 15 % of all trauma-related surgeries in major trauma centers, placing a significant strain on surgical and rehabilitation services [22].

Despite its valuable insights, this study has several limitations. First, as a retrospective analysis, it is subject to documentation bias, particularly concerning helmet use and other safety measures, which were reported in only 9 % of cases. Studies have shown that underreporting of helmet use is common in trauma registries, leading to an inaccurate assessment of safety compliance [23].

Second, the study is limited to a single Level 1 trauma center, which may not fully represent the broader national motorcycle accident trends in Saudi Arabia. Different regions may have varying accident rates, injury patterns, and hospital capacities, affecting generalizability. Additionally, out-of-hospital deaths and cases managed in non-trauma centers were excluded, potentially leading to an underestimation of fatality rates. A study in Southeast Asia found that up to 35 % of motorcycle-related fatalities occur before hospital arrival, suggesting that the true burden of mortality may be higher than reported [24].

Another limitation is the lack of information on other risk factors, such as alcohol or drug use, road conditions, and the involvement of other vehicles in crashes. These factors have been identified in previous research as significant contributors to motorcycle accidents, yet they were not documented in our study [25]. Finally, while the study establishes associations between injury patterns and outcomes, causality cannot be inferred due to the observational nature of the research.

The high rate of surgical intervention emphasizes the need for strong orthopedic and neurosurgical capacity in trauma centers. Prehospital systems also play a crucial role: rapid triage and early recognition of head, chest, and spinal trauma could improve outcomes. With 28.2 % of patients experiencing long-term disability.

Future studies should address the identified limitations to improve the understanding of motorcycle accidents in Saudi Arabia. A nationwide, multi-center study incorporating data from various hospitals and emergency services would provide a more comprehensive overview of motorcycle accident trends and outcomes.

Research on the effectiveness of safety interventions, such as mandatory helmet laws, speed regulations, and motorcycle lane implementations, would be beneficial. Studies have indicated that countries with stricter helmet enforcement and designated motorcycle lanes report lower injury and fatality rates among motorcyclists [26]. Also, we could benefit from public health interventions including school-based safety education, media campaigns, and enhanced enforcement of helmet laws.

Finally, studies analyzing the long-term functional and economic impact of motorcycle injuries would be valuable in assessing the burden on individuals, families, and healthcare systems. Research has shown that long-term disabilities following motorcycle accidents can significantly reduce quality of life and impose financial strain on healthcare systems [27].

By addressing these gaps, future research can contribute to more effective interventions, ultimately reducing motorcycle accident-related morbidity and mortality in Saudi Arabia.

## Conclusion

Motorcycle accidents continue to pose a public health challenge in Saudi Arabia, with young male motorcyclists being the most vulnerable group. The high incidence of fractures and brain injuries emphasizes the importance of improving safety measures to reduce the severity of injuries. Surgical interventions and ICU admissions highlight the substantial healthcare burden, suggesting the need for optimized trauma care systems and targeted preventive measures for high-risk populations such as delivery riders.

## CRediT authorship contribution statement

**Hussam Alhathlol:** Writing – review & editing, Writing – original draft, Visualization, Validation, Supervision, Software, Resources, Project administration, Methodology, Investigation, Formal analysis, Data curation, Conceptualization. **Khalid Alsikhan:** Writing – review & editing, Writing – original draft, Visualization, Methodology, Investigation, Formal analysis, Data curation, Conceptualization. **Turki Alharbi:** Writing – review & editing, Writing – original draft, Visualization, Methodology, Investigation, Data curation, Conceptualization. **Ibrahim Alsamaani:** Writing – review & editing, Writing – original draft, Visualization, Validation, Supervision, Methodology, Investigation. **Ali Alhathloul:** Writing – review & editing, Visualization, Validation, Supervision, Investigation, Data curation, Conceptualization. **Rifan Alyami:** Writing – review & editing, Writing – original draft, Visualization, Validation, Supervision, Methodology, Data curation, Conceptualization.

## Ethics approval

Ethical approval for this study was obtained from KAIMRC protocol no. NRR24/080/7.

## Funding sources

The authors received no funding for this publication.

## Declaration of competing interest

The authors declare no conflict of interest related to this study. The research was conducted independently, with no financial support from external sources or competing interests influencing the outcomes of the study. All authors contributed equally to the design, data collection, analysis, and writing of the manuscript.
